# Protein structure analysis of mutations causing inheritable diseases. An e-Science approach with life scientist friendly interfaces

**DOI:** 10.1186/1471-2105-11-548

**Published:** 2010-11-08

**Authors:** Hanka Venselaar, Tim AH te Beek, Remko KP Kuipers, Maarten L Hekkelman, Gert Vriend

**Affiliations:** 1 CMBI, NCMLS, Radboud University Nijmegen Medical Centre, PO Box 9101, 6500 HB Nijmegen, Netherlands; 2 NBIC, Netherlands Bioinformatics Centre, Geert Grooteplein 28, 6525 GA Nijmegen, The Netherlands; 3 Laboratory of Systems and Synthetic Biology, Wageningen University, Dreijenplein 10, 6703 HB Wageningen, The Netherlands; 4 BioProdict, Dreijenplein 10, 6703 HB Wageningen, The Netherlands

## Abstract

**Background:**

Many newly detected point mutations are located in protein-coding regions of the human genome. Knowledge of their effects on the protein's 3D structure provides insight into the protein's mechanism, can aid the design of further experiments, and eventually can lead to the development of new medicines and diagnostic tools.

**Results:**

In this article we describe HOPE, a fully automatic program that analyzes the structural and functional effects of point mutations. HOPE collects information from a wide range of information sources including calculations on the 3D coordinates of the protein by using WHAT IF Web services, sequence annotations from the UniProt database, and predictions by DAS services. Homology models are built with YASARA. Data is stored in a database and used in a decision scheme to identify the effects of a mutation on the protein's 3D structure and function. HOPE builds a report with text, figures, and animations that is easy to use and understandable for (bio)medical researchers.

**Conclusions:**

We tested HOPE by comparing its output to the results of manually performed projects. In all straightforward cases HOPE performed similar to a trained bioinformatician. The use of 3D structures helps optimize the results in terms of reliability and details. HOPE's results are easy to understand and are presented in a way that is attractive for researchers without an extensive bioinformatics background.

## Background

The omics-revolution has led to a rapid increase in detected disease-related human mutations. A considerable fraction of these mutations is located in protein-coding regions of the genome and thus can affect the structure and function of that protein, thereby causing a phenotypic effect. Knowledge of these structural and functional effects can aid the design of further experiments and can eventually lead to the development of better disease diagnostics or even medicines to help cure patients. The analysis of mutations that cause the EEC syndrome, for example, revealed that some patients carry a mutation that disturbs dimerisation of the affected P63 protein [1]. This information has triggered a search for drugs http://www.epistem.eu; [2]). In another case, the study of a mutation in the human hemochromatosis protein (HFE), which causes hereditary hemochromatosis, resulted in new insights that are now being used to develop novel diagnostic methods [3]. These and numerous other examples have highlighted the importance of using heterogeneous data, especially structure information, in the study of human disease-linked protein variants.

The data that can aid our understanding of the underlying mechanism of disease related mutations can range from the protein's three-dimensional (3D) structure to its role in biological pathways, or from information generated by mutagenesis experiments to predicted functional motifs. Collecting all available information related to the protein of interest can be challenging and time-consuming. It is a difficult task to extract exactly those pieces of information that can lead to a conclusion about the effects of a mutation. Several online Web servers exist that offer help to the (bio)medical researcher in predicting these effects. These servers use information from a wide range of sources to reach conclusions about the pathogenicity of a mutation. The PolyPhen server, for example, is widely used by researchers to predict the possible impact of an amino acid substitution on the structure and function of human proteins [4]. PolyPhen combines a subset of the UniProt sequence features, structural information (when available), and multiple sequence alignments in order to draw conclusions about the impact of a mutation [4]. SIFT, on the other hand, bases its mutation analysis purely on a multiple sequence alignment [5]. This server gives probability scores for each amino acid type at the position of interest to separate the harmless mutations from disease-causing ones. The ALAMUT software http://www.interactive-biosoftware.com/ is widely used in human genetics research groups. It focuses on making many forms of software and databases available to their users. The ALAMUT system also automatically calls the PolyPhen Web server as part of its decision process. ALAMUT is not available as a Web server. PolyPhen, Sift and Alamut all have an excellent track-record make existing data accessible for (bio)medical scientist to aid them with the interpretation of mutational effects. We built on their strengths to produce the HOPE software that was written to optimally use the advantages of the novel tools of the e-Science era.

The recent increase in data types and data volumes has gone hand-in-hand with large efforts in bioinformatics that have led to numerous new databases and computational methods, and in this era of e-Science, Web services provide on-demand access to these facilities [6-8]. The development of Web services facilitates the usage of external databases and methods in in-house developed software and eases software maintenance and development by out-sourcing logic to Web services. Web services have a series of advantages for the software developers:

• They save time by reusing program code;

• They tend to always be up-to-date;

• They are executed remotely, which gives access to large amounts of (free) CPU time, thus not overloading the local machine;

• No need to maintain in-house data and software collections.

Web services also have disadvantages:

• Source code of Web services often is not available;

• Web services are not guaranteed to always be available.

**Figure 1:**
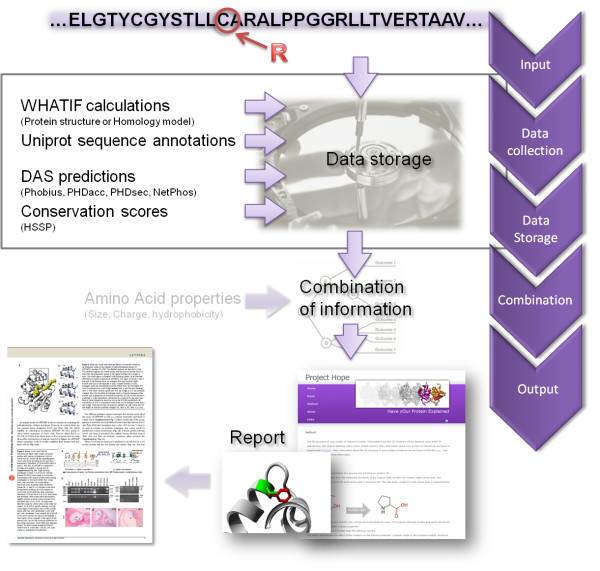
**Overview of HOPE's process flow**. The user submits a sequence and a mutation. HOPE will first collect information from a wide range of information sources. These sources include: WHAT IF for structural calculations on either the PDB file or a homology model that was build by YASARA, HSSP for conservation scores, DAS-servers for sequence-based predictions and Uniprot for sequence annotations. The data is stored in HOPE's information system. The data is combined with the known properties of the amino acids in a decision schedule. The result is a report shown on the HOPE website that will focus on the effect of the submitted mutation on the 3D-structure of the protein. The text and figures can be used in articles and publications.

HOPE (Have (y)Our Protein Explained) is a next-generation web application for automatic mutant analysis. We have designed HOPE to explain the molecular origin of a disease related phenotype caused by mutations in human proteins. In this aspect HOPE resembles the aforementioned systems (PolyPhen, SIFT, ALAMUT). With HOPE we have taken the logical next step in the e-Science era in that the data gathering is done using Web services and DAS servers. Additionally, in HOPE we have taken a protein 3D structure centred approach. HOPE collects information from data sources such as the protein's 3D structure and the UniProt database of well-annotated protein sequences. For each protein this data is stored in a PostgreSQL-based information system. A decision scheme is used to process these data and to predict the effects of the mutation on the 3D structure and the function of the protein. A life-scientist friendly report is produced that explains and illustrates the effects of the mutation. This report is presented using an interface that is designed specifically for the intended user community of human genetics researchers. The report is enriched with figures that illustrate the effects of the mutation, while any residual bioinformatics jargon is linked to our in-house, online dictionary of bioinformatics jargon. The conclusions drawn in the report can be used to design follow-up experiments and eventually can lead to the development of better diagnostics or even medicines. Figure 1 illustrates the major steps of HOPE. We have tested HOPE on a series of mutations that we have previously analyzed manually. In all straightforward cases HOPE performed equally well as a trained protein structure bioinformatician.

***Availability***. The HOPE Web server is freely available on http://www.cmbi.ru.nl/hope/.

## Results and discussion

### Input

**Figure 2:**
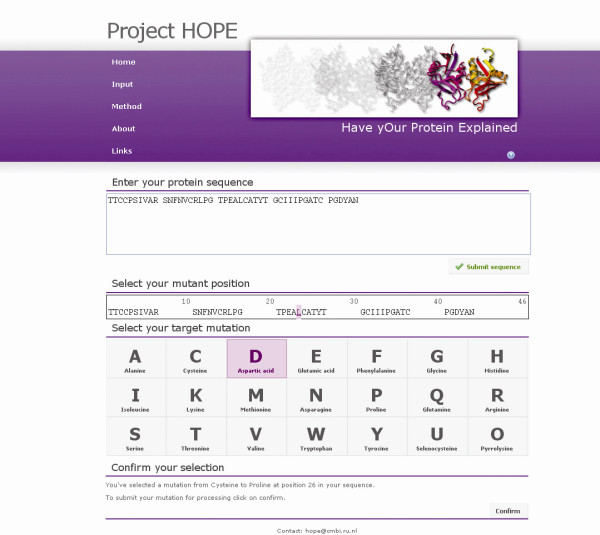
**HOPE's input screen**. The user can submit a sequence of interest and indicate the mutated residue with two simple mouse-clicks. In this example HOPE will analyze a leucine to proline mutation on position 25 of the plant protein Crambin.

The intended users of HOPE are life scientists who neither routinely use protein structures nor bioinformatics in their research. Therefore, both HOPE's input and its results are designed to be intuitive and simple, and all software used will run with default settings so that the user neither needs to set parameters nor needs to read documentation. Actually, the user will not even know which software runs in the background. The interface of HOPE is a website that enables the user to submit a sequence and a mutation. The user can indicate the mutated residue and the new residue type by simple mouse-clicks. Figure 2 shows the input screen, filled with an example protein sequence and a mutation.

### Information retrieval

**Figure 3:**
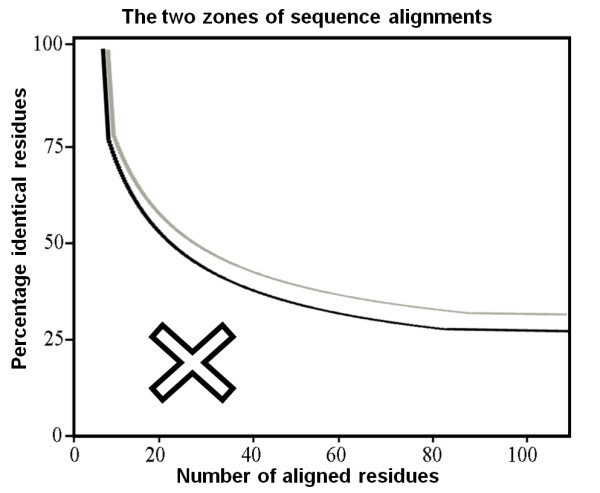
**The two zones of sequence alignment identity that indicate the likelihood of adopting similar structures**. Two aligned sequences are highly likely to have similar folds if their length and percentage sequence identity fall in the region above the threshold (black line). HOPE will build a homology model when the identity between the template and submitted sequence falls in this zone. In case the sequence identity is less than 5% above this threshold (grey line) HOPE will build a model but will also warn that the model is based on a template with low identity. The region below the threshold (indicated with a cross) indicates the zone where inference of structural similarity cannot be made, thus making it difficult to determine if model building will be possible. (Figure free after Sander and Schneider [25]).

HOPE uses the submitted sequence as query for BLAST [9] searches against both the UniProt database [10] and the Protein Data Bank [11]. The search against the UniProt database identifies the protein's UniProt entry and the accession code of the protein, a unique identifier that is used later in the process to obtain DAS-predictions. Alternatively, it is possible to submit this accession code directly. The BLAST search against the PDB is required to find the protein's structure or a possible template for homology modelling. HOPE uses the actual PDB-file when it contains the residue that is to be mutated and when it is 100% identical with the submitted sequence. HOPE identifies among multiple 100% hits the best structure for analysis based on resolution, experimental method, and length of the protein covered in the PDB (a full protein is preferred over a fragment). Nowadays, 20% of the human sequences available from SwissProt have a (partly) known structure and for another 30% a homology model can be build. To be able to build a homology model, the BLAST results should contain the equivalent location of the mutation and the percentage sequence identity should fall above the Sander and Schneider curve shown in Figure 3. Homology modelling is performed using the Twinset version of YASARA which contains an automatic homology modelling script that requires only a sequence as input [12]. The script fully automatically performs the modelling process including sequence alignment, loop building, side chain modelling, and energy minimization. This script was the top contestant in the CASP8 modelling competition in terms of model detail accuracy [13].

The structure of the protein of interest, either a PDB-file or a homology model, is analyzed using WHAT IF Web services [7]. These services can calculate a wide range of structural features (e.g. accessibility, hydrogen bonds, salt bridges, ligand or ion interactions, mutability, variability, etc). When neither a 3D structure nor a possible modelling template is available, HOPE cannot use structural information and will instead base its conclusions only on the sequence related data, and published mutation and variation results.

The UniProt database http://www.uniprot.org/ is used for the retrieval of features that can be mapped on the sequence [14]. This information includes the location of active sites, transmembrane domains, secondary structure, domains, motifs, experimental information, and sequence variants. The UniProt accession code is used to retrieve data from a series of DAS-servers for sequence based predictions such as possible phosphorylation sites. The DAS-servers form a widely used system for biological sequence annotation [15].

The conservation score of the mutated residue is calculated from a HSSP multiple sequence alignment [16].

### Data storage in HOPE

Information obtained from the protein structure or model, the UniProt record, and the DAS-predictions is stored in a protein-specific information system based on the PostgreSQL database system. One new information system is produced for each submitted protein. Differences in the protein sequence might exist between data sources, for example sequences from UniProt often contain the signal peptide while the sequences stored in the PDB tend to lack these residues. Therefore, sequences obtained from different sources are aligned using ClustalW. This enables us to transfer information to the residue of interest without the need to deal with the residue numbering problem that results from these sequence differences. Protein features are stored in the information system on a per-residue basis, and can have one of the following four data-types:

• Contacts: Interaction of the residue with another entity; for example DNA, a metal-ion, a ligand, hydrogen bond, disulfide bond, salt bridge;

• Variable features: Type with a value: for example, accessibility or torsion angle;

• Fixed features: Labels a residue (or stretch of residues) with a feature without a value. This indicates that the residue is located in a domain or motif (for example a residue can be part of the active site or in a transmembrane region);

• Variants: Mutations or other variations in sequence known at this position; for example splice variants, mutagenesis sites, SNPs.

After a user request has triggered the generation of an information system for the protein of interest, the system for this protein is kept on disk for one month just in case the same user (or another user for that matter) requests information about other mutations in the same molecule. After one month every system is thrown away to ensure that conclusions are never based on outdated information. So, there does not really exist a HOPE database as all HOPE's data is, in total agreement with e-Science paradigms, scattered over the internet, and is each time combined upon request.

### Decision scheme

The decision scheme in HOPE uses all collected information combined with known properties of the wild-type and mutated amino acid, such as size, charge, and hydrophobicity, to predict the effect of the mutation on the protein's structure and function. The scheme consists of six parts that each correspond to a paragraph in the output. Each part analyzes the effect of the mutation on one of the following aspects of the residue:

• Contacts. Any interaction with other molecules or atoms, like DNA, ligands, metals, etc, but also hydrogen bonds, disulfide bridges, ionic interactions, etc;

• Structural domain. Any part of the protein with a specific name (and often function), such as domains, motifs, regions, transmembrane domains, repeats, zinc fingers, etc;

• Modifications. Features that do not directly influence the structure of the protein but might influence post-translational processes like phosphorylation.

• Variants. Known polymorphisms, mutagenesis sites, splice variants, etc;

• Conservation: The relative frequency of an amino acid type at each position taken from a multiple sequence alignment.

• Amino acid properties: The differences in the known properties of the wild-type and mutant residue (size, charge, hydrophobicity).

HOPE will produce its conclusions for each of these six aspects separately. For example, a residue can be located in a transmembrane domain and also be important for ligand interaction. HOPE will in this case produce a paragraph about the effect of the mutation on the contacts and a separate paragraph describing the effect of the mutation on the structural location, in this example the transmembrane domain.

Some types of information can be obtained from multiple sources, which are not equally reliable. Experimentally determined features and calculations performed on the 3D coordinates are more likely to be correct than any prediction. For example, transmembrane domains can be predicted by a DAS-server which normally will produce less reliable results than the annotations in UniProt. Therefore, HOPE ranks the information and uses the most accurate source available for its conclusions. WHAT IF calculations are preferred, followed by UniProt annotations, and DAS predictions are used only when neither WHAT IF nor UniProt data are available. In case no information about the mutated residue is found, HOPE will show a conclusion based only on biophysical characteristics between the wild type and mutant amino acid type. The conservation score is obtained either from the HSSP database that holds multiple sequence alignments for all proteins in the PDB, or through the HSSP Web services if a PDB file is not available [16].

### Output

**Figure 4:**
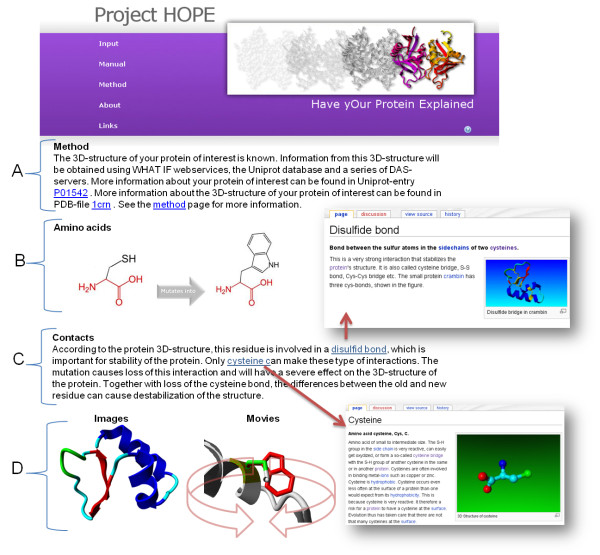
**Example of HOPE's output**. A simplified example of HOPE's output. A) Explanation of the used method (structure, modelling or predictions) and links to the relevant databases. B) Text and pictures that explain the differences between the wild-type and mutant residue. (Text is left out of this figure for clarity.) C) Paragraph of the report explaining the effect of the mutation on contacts made by the residue, a disulfid bond in this case. It contains a link to the wiki-entry "cysteine" and "disulfid bond". D) Images/animations that show the effect of the mutation on the structure.

The report focuses on the effect of the mutation on the 3D-structure, and is aimed at a specific audience in the field of (bio)medical science. It shows the methods used and the sources of the combined information. This can either be an analysis of the real structure or homology model, or a prediction based on the sequence. The results of the mutation analyses are illustrated with figures of the amino acids and, if available, figures and animations of the mutation in the structure. The HOPE output is rather extensive and way too large to put in print, in Figure 4 we just show a small part of one mutation report. A series of examples of HOPE output is available at the "about" section of the HOPE pages.

A HOPE result consists of one HTML page that contains all results. This makes it easy for users to print the results, or to make their own Web-page with HOPE results for long-term storage.

### Test cases

HOPE was validated in a series of collaborations with scientists from different fields of life sciences. Experiences from these real-world examples where used to design and adjust the decision scheme. So far, most mutation studies involved non-sense and missense mutations. Descriptions of these projects can be found at the HOPE website. The resulting reports often contain a molecular explanation of the observed phenotype that can suggest further experiments. The majority of these projects included the building of a homology model as in most cases no 3D-structure of the protein of interest was available.

We also validated HOPE's conclusions by comparing them with the output of PolyPhen and SIFT. Even though it is very difficult to compare the results from PolyPhen, SIFT, and HOPE, we can still draw a few general conclusions, that will be elaborated on in the following paragraphs.

### Structure adds value

The use of a protein's 3D-structure or homology model increases the prediction quality in terms of reliability and detail. The possibility offered by the YASARA software to fully automatically build high quality homology models increases the number of sequences for which HOPE can use structure data. The protein structure, either a PDB-file or a homology model, can reveal information that currently cannot be predicted accurately from sequence alone, such as ionic interactions, ligand-contacts, etc.

The value of the extra information that HOPE can extract from a protein's structure or model is illustrated, for example, by the L320P and L347P mutations in ESRBB (see the "about" section of the HOPE website). All Web servers correctly predict the effect of these mutations as damaging for the protein. However, HOPE completes the story by an extensive explanation of the disturbing effect of prolines on alpha-helices. In cases for which no 3D structure data is available, the three Web servers seem to perform similarly albeit that Polyphen's output often tends to be scarce and a bit cryptic and SIFT's output is limited to conservation scores.

### Biomedicist understandable results

HOPE's interface was designed especially for users that work in the (bio)medical sciences. Instead of displaying data in the form of detailed tables and numerical values, HOPE writes human readable reports that explain the structural and functional effects of the mutation, and illustrates this with figures and animations. When other Web servers list the effects of a mutation as "*Hydrophobicity change at buried site; normed accessibility: 0.00, hydrophobicity change: -2.7*". HOPE will instead report that "*the mutation introduces a less hydrophobic residue in the core of the protein which can destabilize the structure"*. Many more examples of HOPE's readable output can be found at the "about" section of the HOPE website. HOPE's comprehensibility is improved by the Help-function that links difficult bioinformatics keywords to our own in-house dictionary based on Wikipedia's software. In this dictionary the user can find text, illustrations, and sometimes a short video-clip that explains the keyword.

## Conclusions

Upon running 24 test cases, listed on the website, we realised that the present version of HOPE is useful and reliable in analysing point mutations. The next generation of HOPE will, however, need to reach a higher level of data integration to address more complicated cases. Some answer might be found only by combining the calculations with literature data and general knowledge of the protein's structure function relations. For example, PolyPhen predicts the N255D mutation in Kv1.1 (discussed in [17]) as being benign, while SIFT shows that this residue is 100% conserved. Combination of the conservation information with the fact that this residue is located in the voltage sensor of the channel can result in the hypothesis that the mutation disturbs the channel's voltage sensing mechanism. Such conclusions are still beyond the capabilities of today's Web servers, but the software design of HOPE will one day allow us to introduce the features needed to deal which these more complicated cases.

HOPE is an example of the new way of doing data- and software-intensive research in the era of eScience. Nowadays, the ongoing developments in experimental techniques like high-throughput sequencing will continue to produce large amounts of data and will therefore demand new, further automated approaches towards the analysis of these data. The eScience approach used will allow us to easily extend HOPE with more Web services, data sources, and DAS predictions when these become available. In the years to come HOPE can be extended with the possibility to analyze double-mutants, to quantitatively score the structural effects of the mutation and thereby provide the possibility to automatically rank candidate mutations that are the result of a sequence project, or to further improve the already user-friendly HOPE user interface.

## Methods

**Figure 5:**
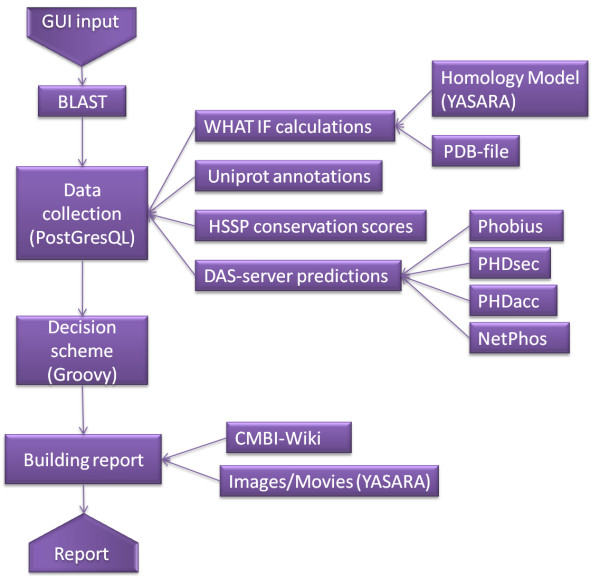
**Detailed overview of HOPE's components**. HOPE's input consists of the sequence and the mutation. The sequence is used for a BLAST search against the databases. Using the accession code (and PDB-file if available) HOPE can collect information from a series of information sources: WHAT IF calculations on the PDB-file or homology model built by YASARA, annotations in the Uniprot database, HSSP conservation scores and sequence-based predictions by DAS-servers. The information is combined in a decision scheme and a report is generated. This report is illustrated with pictures and animations and difficult keywords are linked to our own online dictionary.

The HOPE system is schematically shown in Figure 5. The individual elements of his schema are described in the remainder of this section.

The HOPE website is implemented using the Wicket http://wicket.apache.org/ web framework, which allows us to provide a fluent and responsive user experience. The web application is deployed on the GlassFish web application container https://glassfish.dev.java.net/.

HOPE obtains information from different sources beyond our control. Therefore, the data gathering is set up as fail-safe as possible to handle service unavailability. Data is cached to speed up the process, reduce dependencies and to put less strain on external resources. The data-retention time is 30 days, after which time the data is renewed at the moment someone runs an analysis on the same sequence. The database scheme (available at the "about" section of the HOPE pages) is the result of an iterative design process using both Java and Hibernate to manage all data and to create the database tables. The database engine is PostgreSQL version 8.4.

The MRS BLAST version 4 Web service is used for most database searches with an e-value cut-off of 1e-5 and the low-complexity filter switched off [18]. This Web service http://mrs.cmbi.ru.nl/mrsws/blast/wsdl is backed by an in-house implementation of the standard BLAST algorithm. ClustalW version 2.0.10 is used for sequence alignments [19]. ClustalW is also offered as a Web service through MRS http://mrs.cmbi.ru.nl/mrsws/clustal/wsdl.

WHAT IF Web services, accessible via http://wiws.cmbi.ru.nl/wsdl/, are used to calculate secondary structure (using DSSP [20]), accessibility values, structural fits of mutations, contacts with ligands or ions, salt bridges, disulfide bridges, and hydrogen bonds [21]. These calculations are performed either on the deposited PDB structure, or a homology model. Homology modelling is performed fully automatically using a locally installed WHAT IF & YASARA Twinset [12,13]. This installation runs on a separate server, and is controlled through a Perl CGI script.

Sequence annotations are obtained from the UniProt database http://www.uniprot.org/[14] XML records. The obtained information includes sequence features such as active site, motifs, domains, variants and binding sites.

Conservation scores are obtained from HSSP using the Web service for which the WSDL is available at http://mrs.cmbi.ru.nl/hsspsoap/wsdl. When a PDB deposited structure is available, the pre-calculated HSSP scores maintained at the CMBI are used. In case a homology model is available a DSSP file is generated for the homology model, which in turn is used to create a HSSP file. In case no structure or model is available, a HSSP file is generated using only the user sequence.

Distributed Annotation (DAS) servers [15,22] are used to obtain predictions regarding transmembrane regions by Phobius [23], accessibilities by PHDacc [24], secondary structure by PHDsec [24], and phosphorylation sites by NetPhos [22].

The decision scheme is implemented in Groovy, a dynamic language that runs on the Java Virtual Machine http://groovy.codehaus.org/. The simple Groovy language enables other users to design their own decision schemes and run a specific version of HOPE for their own purposes. The decision scheme is divided into separate branches targeted towards certain aspects of the mutant analysis, each producing a paragraph or sub-report. The decision scheme logic is separated from the phrases used to compose the report, for a cleaner separation in code and to allow for internationalization.

The HOPE report is presented on a self-contained webpage, allowing the user to save the page without breaking links and images. The user can bookmark the URL to perform the same mutant analysis at a later point in time, incorporating any newly available data. The output web pages are intended to be free from bioinformatics jargon. An online dictionary based on MediaWiki's software http://www.mediawiki.org/ is used to explain bioinformatics-specific terms. JavaScript is used to link keywords on the webpage to articles present in the local MediaWiki instance. This functionality is available at any time via the omni-present blue help-button. Images and movies in the report are generated using the YASARA & WHAT IF Twinset.

## Availability and Requirements

The full description of the design and implementation of the HOPE server is available from the "about" section of the HOPE pages. HOPE can be used freely and no licenses are required. The source code has been made open source and can be freely obtained from the HOPE website. HOPE uses Java, Groovy, and PostgreSQL; it has been implemented on a Linux system while care has been taken to avoid system dependencies.

## Authors' contributions

HV was responsible for the biological implementation and validation of decision tree, and for the Wiki and the GUIs. TB designed and implemented the HOPE pipeline and most major elements; RK implemented the decision tree. MH and GV provided the WHAT IF Web services. HV and GV supervised the project. All authors read and approved the final manuscript.
